# Successful outcomes of endoscopic lithotripsy in completely bedridden patients with symptomatic urinary calculi

**DOI:** 10.1038/s41598-020-65807-2

**Published:** 2020-06-01

**Authors:** Yuma Waseda, Ryoji Takazawa, Masaki Kobayashi, Satoshi Yoshida, Yusuke Uchida, Yusuke Kohno, Toshihiko Tsujii

**Affiliations:** Department of Urology, Kidney Stone Center, Tokyo Metropolitan Ohtsuka Hospital, 2-8-1 Minami-Ohtsuka, Toshima-ku Tokyo, Japan

**Keywords:** Renal calculi, Urinary tract infection, Urinary tract obstruction, Urology

## Abstract

Due to the aging population, the number of completely bedridden individuals is expected to increase, and such individuals are at high risk of developing urinary calculi. This retrospective study included 32 consecutive bedridden patients, who had undergone endoscopic lithotripsy between 2010 and 2019, and aimed to identify the treatment outcomes of endoscopic lithotripsy for bedridden patients. A total of 45 endoscopic lithotripsies were performed to treat stones (median cumulative diameter, 24 mm). The stone-free rate (SFR) < 4 mm and complete SFR (0 mm) were achieved in 81% and 63% of patients, respectively. Postoperatively, 10 patients (22%) developed symptoms of systemic inflammatory response syndrome, and three patients (7%) had bloodstream infections. Except for one patient (3%) having a retained ureteral stent ultimately died from septic shock, drainage tube-free discharge was achieved in all patients. The 2-year cumulative incidence of stone-related events, and overall mortality rate, were 18% and 27%, respectively. Endoscopic lithotripsy is well tolerated and is associated with a high success rate, even with severe comorbidities and a relatively large stone burden. Physicians should consider performing endoscopic lithotripsy in bedridden patients with symptomatic urinary calculi regardless of their relatively short life expectancy and the remote risk of perioperative mortality.

## Introduction

The global proportion of people aged ≥65 years is expected to increase from 9% (2018) to 16% by 2050^1^. In Japan, it reached 25% in 2013 and is expected to increase to 30% by 2025^2^. Moreover, the number of people needing long-term care is increasing rapidly, and the number of people requiring long-term care accounted for 4.9% of the total population in 2016^3^. The main causes of disability in patients are stroke followed by dementia. Globally, there have been notable increases in people experiencing strokes, as well as stroke survivors^4^. As the world’s population ages and the number of people with stroke sequelae increases, the number of completely bedridden individuals is also expected to increase.

Extended bed rest is a high risk factor in the development of urinary calculi from hypercalciuria due to immobilization^5,6^. Calculi occasionally cause colic pain, gross hematuria, and acute obstructive pyelonephritis, which has the greatest mortality rate of the urinary tract infections. Acute obstructive pyelonephritis sometimes necessitates an emergency urinary diversion by percutaneous nephrostomy or retrograde ureteral stenting. After managing pyelonephritis, an intervention for the causative stone is considered. Physicians are often hesitant to consider stone removal surgery in patients with a poor general health condition due to a short life expectancy^7–9^, the restrictions on surgical positioning, and the potential for severe complications^10,11^. Therefore, such patients need to undergo periodic drainage tube replacement after stone-related events. However, long-term placement of a drainage tube corresponds to complications such as stone formation and urinary tract obstruction or infection. Surgical intervention for stone removal in completely bedridden patients is still controversial. Therefore, this study aimed to identify treatment outcomes of endoscopic lithotripsy and, for the first time, to propose proper treatment strategies in a limited cohort of bedridden patients with symptomatic urinary calculi.

## Results

### Patients demographics

We included 32 consecutive bedridden patients with symptomatic urinary calculi who received a total of 45 endoscopic lithotripsies. Patient demographics and stone characteristics are summarized in Table 1. A drainage tube was inserted in 24 patients (75%) prior to the first procedure. Thirteen patients (41%) were referred to our hospital for endoscopic lithotripsy after insertion of the drainage tube for the management of obstructive pyelonephritis. Of these 13 patients, 12 patients were inserted ureteral stents, and four (13%) were referred due to challenges during periodical exchange such as retained encrusted ureteral stents or recurrent fever.

**Table 1:** Patient demographics and stone characteristics.

Values		
Gender:		
Female	17	(53%)
Male	15	(47%)
Age, years	76	(32–93)
Body-mass index, kg/m^2^	18.5	(12.7–31.6)
Antiplatelets/anticoagulant drugs	6	(19%)
Parenteral nutrition (enteral/intravenous)	10	(31%)
Tracheostomy tube	5	(16%)
Limb contracture with position limit	14	(44%)
The major underlying disorders		
Stroke	16	(50%)
Neurological disease	5	(16%)
Advanced medical disease	7	(22%)
Senile deterioration	4	(13%)
The reason of surgical intervention		
Obstructive pyelonephritis	27	(84%)
Exacerbation of renal function	1	(3%)
Gross hematuria/Back pain	4	(13%)
Number of stones		
1	13	(41%)
≧2	19	(59%)
Staghorn calculi	7	(22%)
Stone position of targeted stone		
Renal	15	(47%)
Ureter	7	(22%)
Both of renal and ureter	10	(31%)
Laterality of targeted stone		
Left	15	(47%)
Right	14	(44%)
Bilateral	3	(9%)
Highest CT-attenuation value, Hounsfield unit	869	(155–1708)
Index stone diameter, mm	16	(5–78)
Cumulative stone diameter, mm	24	(5–122)
Stone composition		
Calcium oxalate	7	(22%)
Struvite	12	(38%)
Uric acid	1	(3%)
Calcium phosphate	8	(25%)
not analyzed	4	(13%)
Positive preoperative bladder urine culture	28	(88%)
Preoperative drainage tube		
Ureteral stent alone	14	(44%)
Nephrostomy alone	7	(22%)
Both of stent and nephrostomy	3	(9%)
None	8	(25%)
Frequencies of endoscopic lithotripsy		
1	24	(75%)
2	4	(13%)
3	3	(9%)
4	1	(3%)
Operative time, minutes	108	(25–228)
Duration of drainage tube placement, days		
Initial placement - surgery	28	(8–728)
The latest exchange - surgery	27	(4–112)
Postoperative hospitalization, days	11	(2–27)
SFR		
Complete (=0 mm)	20	(63%)
Less than 4 mm	26	(81%)
Observation period, month	15	(0–93)

Categorial variables are shown as number of patients. Continuous variables are shown as median (full range).

CT = Computed tomography; SFR = Stone-free rate.

### Stone characteristics and treatment outcomes

A total of 45 surgical treatments were performed by ureteroscopy (URS), percutaneous nephrolithotomy (PCNL), or both in 33 (73%), 7 (16%), and 5 (11%) cases, respectively. Staged procedures were actually performed in 8 patients (25%), although they were planned in 13 patients (41%) with cumulative stone diameter over 30 mm. The subsequent surgery was generally performed a week after the previous surgery. In total 33 URS cases, eight cases were performed for ureteral stones and 25 cases for renal stones. An average of 1.4 (1–4) procedures/patient was performed to render patients stone and drainage tube-free. Lithotripsies for contralateral side were performed in 3 patients (9%) as a protection against future stone-related events. One patient received bilateral lithotripsies at one session, and other two patients received contralateral procedures at separate sessions. One patient with bilateral renal staghorn calculi of almost 50 mm in size for each side needed a total of four procedures; received two for each side, and achieved completely stone-free status.

### Stone-free rate (SFR) and drainage tube-free discharge rate

The overall SFR < 4 mm and complete SFR (0 mm) were 81% and 63%, respectively. All of the 12 patients (37%) who had residual stones were performed lithotripsy for renal stones, and 5 patients (16%) underwent staged procedures. The median size of residual stones was 4 (2–9) mm, and they were mainly located in the lower calix. One patient who had a retained ureteral stent ultimately died due to septic shock two days after the operation. Except for the patient who died, drainage tube-free discharge was achieved in all patients.

### Cumulative recurrence of stone-related events

**Figure 1:**
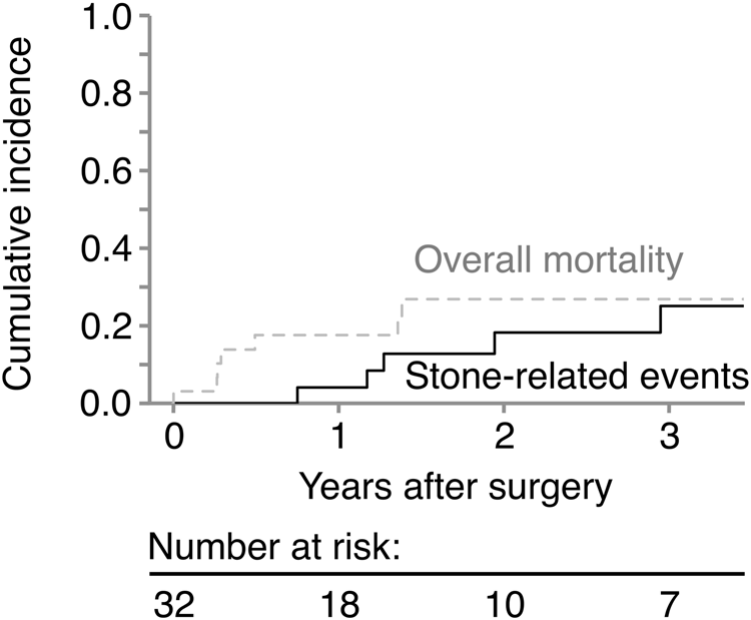
The cumulative incidence of stone-related events (straight line) and death (dashed line).

**Figure 2:**
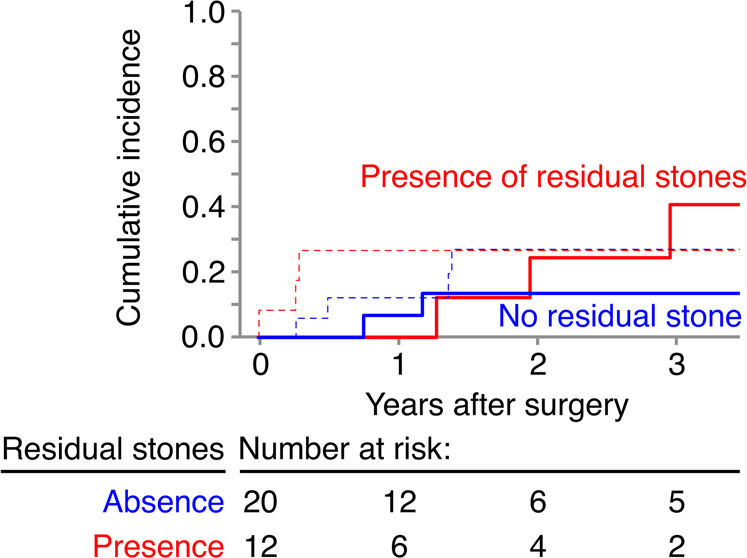
The cumulative incidence of stone-related events (straight line) and death (dashed line) based on the presence or absence of residual stones. The 2-year incidence of stone-related events in patients without residual stones (0 mm; blue straight line) was lower than that in patients with residual stones (red straight line) (13.5% vs 24.4%, p = 0.359).

During a median follow-up period of 15 (0–93) months, 5 patients (16%) experienced a recurrence of stone-related events and 7 patients (22%) had died. Except for the 1 patient who died after operation, the other 6 patients died from unrelated causes within 1.5 years of discharge. According to the competing risk model, the 2-year cumulative incidence of stone-related events and overall mortality rate were 18.3% and 26.9%, respectively (Fig. 1). Although the 2-year cumulative incidence of stone-related events in patients without residual stones was lower than that in patients with residual stones, the difference was not significant (13.5% and 24.4%, respectively; p = 0.359) (Fig. 2).

### Risk factor analysis

Postoperatively, 10 patients (22%) developed symptoms of systemic inflammatory response syndrome (SIRS). Three (7%) had bloodstream infection (BSI), and 1 patient (2%) had died. All incidences of SIRS had occurred after the initial endoscopic lithotripsy. Therefore, we performed risk analyses for SIRS (Table 2) and BSI (Table 3) of only 32 cases of the initial endoscopic procedure. Although larger cumulative stone diameter was a marginally significant factor for SIRS (p = 0.066), it was inversely related to BSI (p = 0.026). BSI was unevenly distributed, and its occurrence was determined by the following 6 necessary conditions: Age>75 years, preceding obstructive pyelonephritis, positive preoperative bladder urine culture, preoperative stent placement, targeted renal stone, and treatment by URS alone. Conversely, BSI did not occur in patients who had a nephrostomy tube at the time of surgery.

**Table 2:** Comparison of clinical characteristics according to SIRS at 32 initial treatments.

	SIRS	non-SIRS	p value
	n = 10	n = 22	
Gender (Male: Female)	4 (40%):6 (60%)	11 (50%):11 (50%)	0.712
Age over 75 (Yes: No)	4 (40%):6 (60%)	13 (59%):9 (41%)	0.450
Body mass index (kg/m^2^)	18 (17–32)	19 (13–28)	0.573
Antecedent obstructive pyelonephritis (Yes: No)	7 (70%):3 (30%)	20 (91%):2 (9%)	0.293
Target to renal stone (Yes: No)	10 (100%):0 (0%)	15 (68%):7 (32%)	0.069
URS alone (Yes: No)	6 (60%):4 (40%)	20 (91%):2 (9%)	0.060
Index stone diameter, mm	24 (5–78)	14 (5–24)	0.065
Cumulative stone diameter, mm	31 (6–122)	22 (5–62)	0.066
Highest CT-attenuation value, Hounsfield unit	1144 (374–1372)	813 (155–1708)	0.484
Struvite calculi (Yes: No)	5 (50%):5 (50%)	7 (39%):11 (61%)	0.698
Preoperative bladder urine culture (Positive: Negative)	9 (90%):1 (10%)	19 (86%):3 (14%)	1.000
Renal pelvic urine culture (Positive: Negative)	6 (60%):4 (40%)	9 (41%):13 (59%)	0.450
Stone culture (Positive: Negative)	5 (50%):5 (50%)	5 (28%):13 (72%)	0.412
Operation time, min	114 (36–181)	81 (25–220)	0.156
Preoperative stent (Yes: No)	7 (70%):3 (30%)	10 (45%):12 (55%)	0.265
Preoperative nephrostomy (Yes: No)	3 (30%):7 (70%)	7 (32%):15 (68%)	1.000
Duration of drainage tube placement, days			
Initial placement - surgery	54 (17–473)	27 (8–728)	0.945
The latest replacement - surgery	35 (17–112)	26 (4–68)	0.135

Categorial variables are shown as number of patients. Continuous variables are shown as median (full range).

SIRS = Systemic inflammatory response syndrome; URS = Ureteroscopy; CT = Computed tomography.

**Table 3:** Comparison of clinical characteristics according to bloodstream infection at 10 patients with SIRS.

	Bloodstream infection		p value
	Presence	Absence	
	n = 3	n = 7	
Gender (Male: Female)	1 (33%):2 (67%)	3 (43%):4 (57%)	1.000
Age over 75 (Yes: No)	3 (100%):0 (0%)	1 (14%):6 (86%)	0.033
Body mass index (kg/m^2^)	20 (18–26)	17 (17–32)	0.764
Antecedent obstructive pyelonephritis (Yes: No)	3 (100%):0 (0%)	4 (57%):3 (43%)	0.475
Target to renal stone (Yes: No)	3 (100%):0 (0%)	7 (100%):0 (0%)	—
URS alone (Yes: No)	3 (100%):0 (0%)	3 (43%):4 (57%)	0.200
Index stone diameter, mm	6 (5–33)	27 (11–78)	0.199
Cumulative stone diameter, mm	15 (6–33)	78 (21–122)	0.026
Highest CT-attenuation value, Hounsfield unit	844 (374–1369)	1219 (629–1372)	0.424
Struvite calculi (Yes: No)	1 (33%):2 (67%)	4 (57%):3 (43%)	1.000
Preoperative bladder urine culture (Positive: Negative)	3 (100%) :0 (0%)	6 (86%):1 (14%)	1.000
Renal pelvic urine culture (Positive: Negative)	1 (33%):2 (67%)	5 (71%):2 (29%)	0.500
Stone culture (Positive: Negative)	1 (33%):2 (67%)	4 (57%):3 (43%)	1.000
Operation time, min	74 (40–141)	115 (36–181)	0.345
Preoperative stent (Yes: No)	3 (100%):0 (0%)	4 (57%):3 (43%)	0.475
Preoperative nephrostomy (Yes: No)	0 (0%):3 (100%)	3 (43%):4 (57%)	0.475
Duration of drainage tube placement, days			
Initial placement - surgery	112 (19–135)	28 (17–473)	0.783
The latest replacement - surgery	42 (19–112)	28 (17–108)	0.829

Categorial variables are shown as number of patients. Continuous variables are shown as median (full range).

SIRS = Systemic inflammatory response syndrome; URS = Ureteroscopy; CT = Computed tomography.

## Discussion

In the current study, endoscopic lithotripsy was well tolerated in completely bedridden patients with symptomatic urinary calculi and was associated with good success rate and low complication rate, despite the presence of severe comorbidities. Unfortunately, one patient died following endoscopic lithotripsy; however, the patient had a retained stent due to stone formation. Ureteral stents have potential complications such as encrustation and stone formation, and can subsequently result in mortality^12^. Encrustation rates are reported as 9–27% at <6 weeks and 48–57% at 6–12 weeks^13,14^. In this study, endoscopic lithotripsy was performed in 4 of the patients due to drainage tube trouble.

Patients with low functional status have a lower life expectancy because of comorbidities^7–9^. In our study, 6 patients had died from causes unrelated to stones within 1.5 years of discharge. Additionally, the negative impact of low functional status on postoperative infection has been reported^10,11^. The possibility of overtreatment and severe complication can make physicians hesitant about performing endoscopic surgery in such cases. Although observation therapy with periodic drainage tube replacement is a seemingly innocuous strategy, it can lead to stone-related death. Yamashita *et al*. have reported that in patients with performance status 3–4, stone-specific survival for the observation therapy group was 61.3%, although that for nephrolithotomy group was 100%^15^. In our cohort, four patients (13%) ended up being referred due to drainage tube troubles. In particular, a patient with a retained encrusted ureteral stent after periodical replacement over one and a half years resulted in postoperative mortality. Physicians should consider endoscopic lithotripsy for bedridden patients with urinary calculi regardless of the relatively short life expectancy and remote risk of perioperative mortality in these cases^16^.

Extended bed rest causes increased bone resorption, and a marked increase in urinary calcium and phosphate is also a major contributor to stone formation^5,6,17^. There have been few reports on stone removal surgery for patients with poor functional status. Complete SFR was only 47% in patients with spinal cord injury and a median stone size of 16 mm, with repeated intervention being required in 19% of these patients^18^. In patients with performance status 3–4 and a median stone size of 11.5 mm, SFR < 4 mm was reported to be 50% after PCNL and 87% after URS^15^. In this study, the overall SFR was 63% (complete) and 81% (<4 mm) when using a staged procedure, for 25% of the patients. This outcome is acceptable considering the challenges associated with these cases, such as a relatively large stone burden, restrictions on surgical position, and severe comorbidities. The 2-year recurrence rate of stone-related events was 18% in this study. Interestingly, the recurrence rate was lower in those who had achieved complete stone-free status, although the difference was not statistically significant. Therefore, to reduce the recurrence rate, the achievement of complete stone-free status might be important. Since the risk of repeat intervention would be lower than that of the initial treatment, planning a staged procedure to achieve complete stone-free status should be prioritized.

Infectious complications are the most feared risks following endoscopic lithotripsy. Several studies have attempted to identify factors regarding the patient, stones, and intraoperative parameters that can lead to fever, SIRS, or sepsis following endoscopic lithotripsy. For better understanding of these risks, analyses of a cohort of bedridden patients would be essential, because increased vulnerability can make the risk factors more explicit. Bedridden status corresponds to frailty, which is independently associated with adverse outcomes^19^. In the current study, the incidence of SIRS was actually higher (22%) than that of our entire cohort (9.6%) reported previously^20^. Moreover, BSI was considered to be another representative index, since it would appear during an infective exacerbation. We highlighted 6 necessary conditions for BSI. In planning lithotripsy for targeted stones, it would be preferable not to satisfy following 3 therapeutic aspects: targeted renal stones, preoperatively placed ureteral stent, and treatment by URS alone. These factors are in agreement with those in a previous report demonstrating that intrarenal pressure during endourological procedures is a risk factor of postoperative infection^21^. Conversely, BSI did not occur in patients who had a nephrostomy tube at the time of surgery. The optimal drainage for obstructive pyelonephritis has not been established. For surgical decompression of infected hydronephrosis, percutaneous nephrostomy might be superior to ureteral stents^22^. Moreover, percutaneous nephrostomy can help in maintaining intraoperative irrigation and can prevent high renal pelvic pressure. Percutaneous nephrostomy would benefit completely bedridden patients by establishing the reliable drainage route.

This study has several limitations. First, it was a single-center study with a relatively small cohort. The small sample size and event rate limited our ability to statistically determine the risk factors. A multi-institutional study with a large patient cohort is required for validation of the outcomes. Second, this study included patients who had received lithotomy after initial emergency drainage for acute obstructive pyelonephritis, and did not include a conservatively treated control group. This was because all patients were treated by endoscopic lithotripsy, even when patients had severe comorbidities, and no patients desired periodic drainage tube replacement in our hospital. The affordable treatment results were all the more significant because there was no bias in treatment selection. Although a comparison with a control group may be required, it is impractical in studies involving such patients with various complications. In addition, it is unethical to put patients in potentially life-threatening situations in a randomized trial. Thirdly, we believe that the drainage tube-free status achieved from surgeries would provide relief from future tube-related complications, onerous tube care, and routine hospital visits. A risk–benefit assessment, including the quality of life of both patients and their family, would be the next step to ensure the best treatment decisions. Despite these limitations, we were able to demonstrate that active intervention can be safely performed under careful management, with the exception of a patient with retained encrusted ureteral stent. Especially, in the case of completely bedridden patients with renal calculi, establishing the reliable drainage route by percutaneous nephrostomy would be a useful treatment strategy to prevent severe postoperative infection.

## Conclusions

Endoscopic lithotripsy is well tolerated in completely bedridden patients and is associated with a high success rate, even when there are severe comorbidities and a relatively large stone burden. Physicians should consider performing endoscopic lithotripsy on bedridden patients with symptomatic urinary calculi regardless of their short life expectancy and remote risk of perioperative mortality. Performing percutaneous nephrostomy as the drainage route for bedridden patients having renal stone at the time of acute pyelonephritis, or at least performing active stone removal, would be useful in preventing severe postoperative infectious complications.

## Methods

### Patients selection and data collection

Between April 2010 and March 2019, 1092 cases of endoscopic lithotripsy were performed at Kidney Stone Center, Tokyo Metropolitan Ohtsuka Hospital. This retrospective study included 32 consecutive patients with symptomatic urinary calculi who were completely disabled and confined to bed. Bedridden patients with silent stone were not included. During this survey period, no bedridden patients with symptomatic urinary calculi were managed conservatively, regardless of comorbidities. Written informed consent was obtained from all participants and/or their surrogates, and this study was approved by the institutional ethical committee of Tokyo Metropolitan Ohtsuka Hospital (approval number #2015–15). All procedures were performed in accordance with the relevant regulations and guidelines.

Patient information including age, sex, body mass index, underlying comorbidities, antecedent acute pyelonephritis, and presence or absence of urinary drainage prior to endoscopic lithotripsy was collected retrospectively from medical records. Urinary drainage was defined as placement of a nephrostomy tube or ureteral stent, and the duration from placement of urinary drainage to surgery was recorded. We generally selected establishing nephrostomy at the time of pyelonephritis as long as there was hydronephrosis in patients with larger renal stones over 20 mm, since PCNL was considered as first-line choice. In patients with prolonged ureteral stent dwelling time over 2 months, ureteral stents were replaced before surgery excluding retained encrusted ureteral stents. Stone characteristics such as location and size were determined by non-contrast computed tomography (NCCT). The index stone in each patient was defined as the largest impacted stone of ureter, or as the largest renal stone if there were multiple renal stones. The index stone diameter was defined as the largest diameter of the index stone, and the cumulative stone diameter was defined as the sum of the maximum diameter of each stone. We measured the highest CT-attenuation value of the region of interest, which was traced to maximally cover the index stone. Stone composition groups were determined according to the most prominent crystal, except for struvite calculi, when any amount of struvite was identified.

A positive preoperative bladder urine culture result was defined as ≥10,000 colony forming units/ml. Antibiotic sensitivity was also analyzed. Pathogen-specific antibiotic therapy was administered for an adequate duration as prescribed by the treating urologists. Specimens for renal pelvic urine cultures and stone cultures were obtained intraoperatively.

### URS and PCNL

Surgeries were generally performed under general anesthesia, and 5 patients (16%) received lumbar anesthesia. URS was performed in the lithotomy position using 6.5/8.5-Fr semirigid ureteroscope (Richard Wolf, Knittlingen, Germany) and 7.95–8.4 Fr flexible scope (URF-P5, URF-P6; Olympus, Tokyo, Japan, or Flex-X2; Karl Storz, Tuttlingen, Germany). Stones were fragmented using holmium:YAG (yttrium-aluminum-garnet) laser (VersaPulse; Lumenis, Yokneam, Israel and Litho; Quanta System, Milan, Italy) at 5 Hz, 1.2 J with a 200-μm laser fiber. Ureteral access sheaths (12/14 or 10/12 Fr ReTrace; Coloplast, Humlebaek, Denmark) were used in all flexible ureteroscopy cases. 6 Fr ureteral stents were placed after operation in all cases. For PCNL, the patients were placed in the modified supine Valdivia position. Lithotripsy by holmium:YAG laser at 5–12 Hz, 1.2 J with a 550-μm laser fiber or a pneumatic lithotripter (Swiss LithoClast 2; EMS, Nyon, Switzerland) was performed by a 12-Fr miniature nephroscope (Karl Storz, Tuttlingen, Germany) through 16- or 18-Fr tracts; 14- or 16-Fr nephrostomy tubes were inserted in all percutaneous tracts.

PCNL was considered as first-line choice in patients with larger renal stones over 20 mm, and simultaneous URS as retrograde intrarenal surgery was used to render patients stone-free at one time. When patients had large renal stones at the contralateral side of the causative stone, bilateral surgery was performed. Patients with high stone burden such as cumulative stone diameter over 30 mm undergo repeat endoscopic lithotripsy as a planned staged procedure to avoid complications associated with a longer operation time.

### Follow-up

Drainage tube removal was generally done on postoperative day 7. Drainage tube removal and stone-free status evaluation were performed during hospitalization. SFR, defined as the complete absence of stones or residual fragments <4 mm at the affected site, was mostly determined using NCCT, and kidney–ureter–bladder radiography was used in others. The diagnostic criteria for SIRS were as follows: i) body temperature>38 °C or <36 °C; ii) heart rate>90 beats/minute or partial pressure of CO_2_ < 32 mm Hg; iii) respiratory rate>20 breaths/minute; and iv) white blood cell count>12,000/mm^3^ or <4000/mm^3^_,_ or immature neutrophils>10%. SIRS was determined based on the most adverse physiological/laboratory data measured after operation. In case of prolonged fever>38 °C after surgery, presence of BSI was ascertained from a blood culture. Patients who could return for a follow-up visit were followed every six months, and other patients were followed by telephone to ascertain vital status and the presence of recurrent stone-related events.

### Outcome assessment

The primary outcomes were SFR and drainage tube-free discharge rate. The cumulative recurrence of stone-related events was analyzed using a competing risk model, with death as the competing event. Stone-related events included stone colic, gross hematuria, and pyelonephritis associated with calculi. Furthermore, the SIRS criteria and BSI were adopted as indexes for postoperative infectious complications, and the risk factors associated with these indexes were analyzed. Vital status and recurrence of stone-related events were confirmed from retrospective chart review and telephonic survey.

### Statistical analyses

Associations between clinical variables and infectious complications were determined using the Fisher’s exact test for categorical variables and the Wilcoxon rank sum test for continuous variables. Statistical analyses were performed with SPSS version 20 (SPSS Inc., Chicago, IL, USA) and R v.3.3.1 (R Foundation for Statistical Computing, Vienna, Austria) with cmprsk package v.2.2–7. P < 0.05 was considered statistically significant.
